# Review of *Practical Human Behaviour Change for the Health and Welfare of Animals*

**DOI:** 10.1017/awf.2025.24

**Published:** 2025-04-23

**Authors:** Grace Carroll

**Affiliations:** Lecturer in Animal Behaviour and Welfare, School of Psychology, Queen’s University Belfast, Belfast, Northern Ireland, UK

The publication of this book is very timely. In the last five years, the field of Animal Welfare Science has seen a growing interest in the role of human behaviour in ensuring good animal welfare. Frameworks from psychology are being increasingly used to address various challenges, from identifying barriers to achieving good animal welfare to designing effective behaviour change interventions. This need for interdisciplinarity is reflected in the author, Bronwen Williams’, career path. Williams, a mental health nurse and equine welfare volunteer, has extensive experience with motivational interviewing in both her NHS role and her work with horse owners. The book focuses on motivational interviewing — an approach with strong roots in psychology — which I believe is much needed in animal welfare but remains under-utilised. This book will be particularly useful for those working one-on-one with individuals rather than those addressing the behaviour of large groups of people.

Chapters 1 and 2 set the scene, introducing key concepts and encouraging readers to apply these to their own experiences. One of the book’s strongest aspects is its use of detailed real-world examples, which help illustrate how understanding different perspectives can change the way we interpret behaviour. Williams explains why common strategies for influencing behaviour, such as advising, bribing, or persuading are often ineffective. Instead, this book provides a framework to help those working in animal welfare to support owners and other stakeholders in identifying necessary changes themselves. Chapter 3 introduces the stages of change model, which outlines the journey from precontemplation through to contemplation, preparation, action, and maintenance, where the person has successfully adopted a new behaviour and is working to sustain it over time. Chapter 4 describes motivational interviewing in detail, explaining common pitfalls in clear and accessible language. Chapter 5 focuses on active listening, a critical skill in motivational interviewing. Williams provides practical tips and exercises to help readers assess and improve their listening skills, tailoring their approach depending on the situation — whether simply listening, offering advice, or guiding someone toward their own solutions. From Chapter 6 onwards, the book moves into applied practice, encouraging readers to revisit earlier chapters as needed, particularly Chapter 5 which is central to the book and to the skills development of the reader. Williams presents strategies for working with individuals who are undecided about change, or at the early stages of change. At this stage, Williams emphasises the importance of setting shared agendas and avoiding premature goal-setting. The book outlines practical techniques such as constructing timelines to track the history of a situation, mapping out daily routines to identify problem areas, and using rating scales to assess readiness for change. Rather than imposing solutions, Williams advocates for an approach that encourages individuals to articulate their own understanding of the situation.

As the book progresses, it covers how to support owners through the change process when they have decided they are ready, from preparation for change to action (Chapter 11) and into the maintenance phase (Chapter 12). Chapter 13 focuses on skill development for the reader, while Chapter 14 provides useful templates for exercises that can be used with animal owners, also available for download online as a companion to the book.

Williams is pragmatic about the challenges of changing human behaviour and encourages self-reflection throughout, making this book an accessible and introspective read. While it is primarily aimed at those working directly with people in animal welfare contexts, researchers conducting qualitative studies may also find its approaches useful. The book is well-structured and easy to read, with information presented in manageable sections. Key points are highlighted using bullet points and case studies, avoiding the overwhelm that dense blocks of text can sometimes create. The writing is engaging and practical, making it a valuable resource for those new to motivational interviewing, as well as those seeking to refine their skills. The inclusion of ‘practical’ in the title is well justified — the book delivers on this by providing concrete examples and equipping readers with the tools to apply motivational interviewing effectively in real-world settings. One minor limitation is that the book’s title does not fully convey its content. I initially expected broader behaviour change frameworks to be referred to in the book, though references to these concepts do appear indirectly. In summary, *Practical Human Behaviour Change for the Health and Welfare of Animals* is a highly practical, accessible, and engaging guide, offering a fresh and much-needed perspective on motivational interviewing in the context of animal welfare.

